# Long-Term Histologic Changes in Nasal Mucosa after Total Laryngectomy

**DOI:** 10.1155/2010/640818

**Published:** 2010-06-21

**Authors:** Chaudhary Farqan Riaz

**Affiliations:** Department of ENT, Leicester Royal Infirmary, Infirmary Square, Leicester LE1 5WW, UK

This recently published interesting paper [1] showed statistically significant reduction (*P* < .005) in congestion of inferior turbinate mucosa in 2 groups of post-total laryngectomy patients over a period of time (group 1; less than 12 months, group 2; 12–36 months). I am intrigued as to which statistical test was used to calculate this and the other *P* values, since it was not mentioned in the study.

Also, I wonder what the authors' and readers' opinions are with regards to the time intervals at which biopsies were taken postoperatively. Could the other histological indices have born a statistically significant difference between the groups, where shorter time intervals are to be used (e.g., 3, 6, and 9 months)? The references cited by the authors themselves certainly do not seem to observe any standard established time frames. 

It would be interesting to gauge readers' opinion on these matters.
